# Identification of an interactome network between lncRNAs and miRNAs in thyroid cancer reveals SPTY2D1-AS1 as a new tumor suppressor

**DOI:** 10.1038/s41598-022-11725-4

**Published:** 2022-05-11

**Authors:** Julia Ramírez-Moya, León Wert-Lamas, Adrián Acuña-Ruíz, Alice Fletcher, Carlos Wert-Carvajal, Christopher J. McCabe, Pilar Santisteban, Garcilaso Riesco-Eizaguirre

**Affiliations:** 1 Instituto de Investigaciones Biomédicas “Alberto Sols”, Consejo Superior Investigaciones Científicas, Universidad Autónoma de Madrid (CSIC-UAM), 28029 Madrid, Spain; 2 Centro de Investigación Biomédica en Red de Cáncer (CIBERONC), Instituto de Salud Carlos III (ISCIII), 28029 Madrid, Spain; 3 Institute of Metabolism and Systems Research, University of Birmingham, Birmingham, B152TT UK; 4 Department of Bioengineering and Aerospace Engineering, Universidad Carlos III, 28911 Madrid, Spain; 5 Hospital Universitario de Móstoles, 28223 Madrid, Spain; 6 Endocrinology Molecular Group, Faculty of Medicine, Universidad Francisco de Vitoria, Madrid, Spain

**Keywords:** Cancer genomics, Endocrine cancer, Endocrinology

## Abstract

Thyroid cancer is the most common primary endocrine malignancy in adults and its incidence is rapidly increasing. Long non-coding RNAs (lncRNAs), generally defined as RNA molecules longer than 200 nucleotides with no protein-encoding capacity, are highly tissue-specific molecules that serve important roles in gene regulation through a variety of different mechanisms, including acting as competing endogenous RNAs (ceRNAs) that ‘sponge’ microRNAs (miRNAs). In the present study, using an integrated approach through RNA-sequencing of paired thyroid tumor and non-tumor samples, we have identified an interactome network between lncRNAs and miRNAs and examined the functional consequences in vitro and in vivo of one of such interactions. We have identified a likely operative post-transcriptional regulatory network in which the downregulated lncRNA, SPTY2D1-AS1, is predicted to target the most abundant and upregulated miRNAs in thyroid cancer, particularly miR-221, a well-known oncomiRNA in cancer. Indeed, SPTY2D1-AS1 functions as a potent tumor suppressor in vitro and in vivo, it is downregulated in the most advanced stages of human thyroid cancer, and it seems to block the processing of the primary form of miR-221. Overall, our results link SPTY2D1-AS1 to thyroid cancer progression and highlight the potential use of this lncRNA as a therapeutic target of thyroid cancer.

## Introduction

Thyroid cancer is the most frequent cancer arising from the endocrine system^1^, with an increasing trend in incidence rates in recent decades^2–4^. Thyroid cancer generally has a good prognosis, as most of patients with this disease can be cured with surgery and radioactive iodine treatment. However, a significant number of patients develop metastatic disease refractory to radioactive iodine treatment and their life expectancy is substantially reduced. Based on their histological appearance, thyroid cancers can be categorized as differentiated or undifferentiated cancers. The former includes papillary thyroid carcinoma (PTC)—the most common type of thyroid cancer—and follicular thyroid carcinoma (FTC). The latter includes undifferentiated anaplastic thyroid carcinoma (ATC)—which is the most aggressive histotype^5^. Recent advances in sequencing technology and high-throughput analysis have increased our understanding of the molecular events underlying the etiology of thyroid cancer. There is evidence that a number of genetic events leading to the hyperactivation of both the MAPK and the PI3K/AKT pathways are responsible for the initiation of well-differentiated thyroid cancer, yet the key events leading to the progression and dissemination of the disease remain incompletely understood.

Genomic characterization of many forms of human cancer has revealed that the bulk of the genome is transcribed into non-coding RNAs, whose roles are only now beginning to be understood in detail. In particular, recent efforts to genetically characterize PTC by The Cancer Genome Atlas (TCGA) research network and others^6–9^ have demonstrated that non-coding RNAs are important in PTC initiation and progression. The most widely studied non-coding RNAs are the microRNAs (miRNAs), which are trans-acting, single-stranded RNA molecules of ~ 22 nucleotides that post-transcriptionally regulate gene expression. miRNAs are transcribed by RNA polymerase in the nucleus into primary miRNA (pri-miRNA) transcripts, which are then recognized and cleaved by the nuclear Rnase III enzyme DROSHA, liberating precursor miRNAs (pre-miRNAs) that are exported from the nucleus and further processed by the DICER1 enzyme to form mature miRNAs. Finally, the mature strand is loaded onto the RNA-induced silencing complex (RISC) to guide the complex to complementary target mRNAs for gene silencing. miRNAs are largely deregulated in cancer, and this process of deregulation has been relatively well characterized in thyroid cancer^10,11^. Some specific miRNAs including miR-146b, miR-21 and miR-221 are upregulated during thyroid tumorigenesis and can influence several biological traits, for instance, aggressiveness and cell migration^6,7,12^. miRNAs can thus serve as clinical markers for diagnosis and prognosis; in particular, miR-221 has been described as an important upregulated “oncomiRNA” in many tumor types^13–17^ including thyroid cancer^6^, where it has been shown to promote proliferation and invasion^18,19^, and epithelial-mesenchymal transition^20^. Indeed, miR-221 levels are significantly associated with lymph node metastasis, tumor-node-metastases stage^20^ and PTC recurrence^21^, and are consistently upregulated in different types of thyroid carcinomas^22^. Accordingly, miR-221 has likely a strategic function, and it might serve as a potential therapeutic target and biomarker of malignancy.

How miRNAs are deregulated in cancer has been a subject of intensive investigation. The interplay between miRNAs and transcription factors is likely to be very important in tumor biology, as both share common targets and seem to coordinately regulate complex regulatory networks^23^. Interestingly, other forms of non-coding RNAs, such as long non-coding RNAs (lncRNAs), are also beginning to be appreciated as an important layer of genome regulation. In fact, lncRNAs—typically 200 nucleotides in length—are implicated in numerous biological processes, such as imprinting, epigenetic regulation, nuclear import, cell cycle control, cell differentiation, alternative splicing, RNA decay and transcription^24^. LncRNAs are frequently cell type-specific and alterations in their expression have been observed in several cancer types^25–27^. Thus far, several lncRNAs have been shown to act as oncogenes or tumor suppressors, and they have the potential to be essential players in thyroid cancer biology^24^.

Although miRNAs and lncRNAs are functionally and evolutionary distinct, several levels of interaction are known. Notably, some lncRNAs contain binding sequences for miRNAs, and are able to titrate the amount of free miRNA, reducing their action on mRNA targets. Such lncRNAs are termed “miRNA sponges”, which adds an additional layer of complexity to the miRNA interactome. Because this interactome has yet to be fully explored in thyroid cancer, here we studied the main differentially expressed lncRNAs and their interactions with common differentially expressed miRNAs in PTC. We performed RNA sequencing (RNA-seq) of paired normal and tumor thyroid samples from 8 patients with PTC and analyzed lncRNA–miRNA interactions using two different homology-based algorithms. We identified one downregulated lncRNA, SPTY2D1-AS1 (hereafter, SPTY), which seems to act as a tumor suppressor based on functional in vivo and in vitro studies. We also established that SPTY functions as a sponge for miR-221, one of the most upregulated and oncogenic miRNAs in thyroid cancer. Interestingly, this effect could be mediated by the impairment of miRNA biogenesis and not only through the sponging of the mature strand.

## Results

### Identification of an interactome network between lncRNAs and miRNAs in thyroid cancer reveals SPTY as a downregulated lncRNA predicted to interact with multiple thyroid cancer-related miRNAs

To identify differentially expressed lncRNAs, we re-examined our previous RNA-Seq data^7^ of paired normal and tumor thyroid tissue from 8 patients with PTC, the most common type of thyroid cancer. Using Gencode (v19) as a reference, we identified 32 upregulated (Table 1) and 44 downregulated (Table 2) lncRNAs from the pairwise comparisons.

**Table 1:** Overexpressed lncRNAs (false discovery rate < 0.05; fold change > 2).

lncRNA	Fold-change	P-value	FDR	Mean RPM PTC	Mean RPM normal
LOC100130705	19.3632	1.4343E−18	2.4703E−15	253.3209	8.1834
DCTN1-AS1	15.2411	2.1563E−10	7.2663E−08	44.1498	2.1858
LRP4-AS1	16.7912	3.0847E−10	9.9618E−08	214.4604	10.2615
LOC100131347	3.7826	1.3759E−09	3.8086E−07	113.1143	27.4802
LOC728558	6.3430	9.7635E−08	1.4414E−05	623.4261	88.1128
LOC645513	5.0967	1.3736E−07	1.9357E−05	1740.2532	272.9372
LOC100128593	4.4017	5.8395E−07	6.6165E−05	44.3900	9.6630
RPSAP52	20.0636	1.7698E−06	0.00016831	12.2206	0.0000
RPARP-AS1	2.6555	2.528E−06	0.00022139	96.8944	36.1304
LOC401320	4.0915	5.5897E−06	0.00042894	27.0555	6.4969
LOC100128770	2.4220	1.595E−05	0.00098893	73.6190	29.7520
CCDC148-AS1	5.7849	7.5373E−05	0.00333817	16.2734	2.2981
LOC730102	2.8450	8.167E−05	0.00351657	71.7245	24.3745
GUSBP11	4.1950	0.00010669	0.00428445	24.7328	5.2304
PCED1B-AS1	2.4438	0.00013108	0.0049861	40.1751	16.4914
GRM5-AS1	12.1310	0.00017157	0.00624301	7.4701	0.0000
POM121L10P	2.1790	0.00026406	0.00853805	138.7576	59.8491
LOC100130872	2.3836	0.00026719	0.00859277	97.4886	36.9943
LOC100287042	1.8843	0.00030794	0.00958521	326.8392	169.3835
LOC100507424	3.2131	0.00055525	0.01515313	39.3935	15.5065
MIRLET7DHG	2.1851	0.00068445	0.01742143	285.3785	134.0258
SEC1P	1.8417	0.00093998	0.02165025	400.9397	197.1720
SEPT7P2	9.3661	0.00104191	0.02337279	4.7380	0.0000
PITPNA-AS1	1.6792	0.00128579	0.02737787	391.8366	227.3212
OR7E37P	5.5547	0.00134185	0.02817867	11.4625	1.5201
MKNK1-AS1	1.7463	0.00166233	0.03303561	152.8900	88.6166
HPN-AS1	2.2801	0.00184612	0.03554877	98.8192	42.4373
STARD4-AS1	1.7105	0.00185588	0.03564803	714.3363	393.5151
PSMB8-AS1	1.8856	0.00212766	0.03978389	1770.2184	978.1383
LOC644656	8.0021	0.00244504	0.04407824	4.1915	0.0000
TPRG1-AS2	4.4655	0.00248082	0.04457514	9.5893	1.7188
MALAT1	1.7984	0.00269796	0.04698999	1105.1707	594.9314
POM121L8P	3.1743	0.0027361	0.04746495	23.6705	7.0317

**Table 2:** Downregulated lncRNAs (false discovery rate < 0.05).

lncRNA	Fold-change	P-value	FDR	Mean RPM PTC	Mean RPM normal
PEG3-AS1	0.2327	5.3762E−11	0.0000000	45.7802	192.7842
DIO2-AS1	0.1310	7.4998E−10	0.0000002	28.4134	145.1241
NCAM1-AS1	0.2515	1.8709E−08	0.0000038	24.2869	92.0914
LOC100130238	0.1697	1.4459E−07	0.0000202	4.5598	32.0197
PAX8-AS1	0.3773	1.1676E−06	0.0001168	1168.1150	2900.3187
FAM13A-AS1	0.3803	2.4703E−06	0.0002176	466.3345	1066.4841
LINC00847	0.4159	3.0847E−06	0.0002599	105.6587	244.2889
ADD3-AS1	0.2477	1.0552E−05	0.0006990	6.5854	27.1494
RAD21-AS1	0.2109	4.0159E−05	0.0020277	4.6380	21.5116
**SPTY2D1-AS1**	**0.4200**	**4.2188E−05**	**0.0021028**	**84.2462**	**192.8508**
ST7-AS1	0.3458	5.6163E−05	0.0026542	18.4053	51.6345
KCNQ1OT1	0.4772	7.4542E−05	0.0033153	235.2624	458.0924
SLC25A5-AS1	0.4574	8.9763E−05	0.0037552	75.1143	157.7156
LOC100507346	0.4079	0.00011389	0.0044582	21.4538	52.5575
MYCBP2-AS1	0.3956	0.00016461	0.0060463	52.7146	121.2391
SLC26A4-AS1	0.2892	0.00019439	0.0068637	103.4308	245.7455
PRICKLE2-AS1	0.4765	0.00021314	0.0073380	279.8504	553.9219
ATP1A1OS	0.5428	0.00021786	0.0074547	10,681.5300	18,578.5176
TPTE2P5	0.3222	0.00026334	0.0085380	101.6748	214.4319
STARD7-AS1	0.3053	0.00044844	0.0127546	21.6244	56.6701
LOC100288181	0.3887	0.00056783	0.0154150	26.0891	62.2720
EHHADH-AS1	0.3760	0.00059177	0.0158157	22.2171	56.2585
LEF1-AS1	0.2474	0.00063985	0.0167539	3.3123	16.5520
GLIS3-AS1	0.3479	0.00066141	0.0170876	8.3462	27.3202
RRP7B	0.4605	0.00069406	0.0175804	29.4073	63.3598
TMEM220-AS1	0.3255	0.00076419	0.0187084	8.5117	29.4730
EFCAB14-AS1	0.4287	0.0009818	0.0224468	40.1879	84.7427
LOC255130	0.5712	0.0009869	0.0224969	741.6513	1235.8579
LOC646903	0.1397	0.0010221	0.0230442	0.3673	7.5458
ST7-OT4	0.2869	0.00115751	0.0252711	4.1116	18.8228
MIR3661	0.4715	0.00123036	0.0264885	64.0232	122.9162
CLIP1-AS1	0.1640	0.00124566	0.0267067	2.1418	16.7339
TMPO-AS1	0.4153	0.00125557	0.0268821	14.7127	34.8778
LOC100652999	0.5556	0.00126515	0.0270034	192.5656	321.2917
IQCH-AS1	0.5261	0.00131516	0.0277742	68.3125	127.1111
LOC646736	0.3323	0.00190092	0.0363780	7.1490	24.0098
SNRK-AS1	0.6022	0.0019717	0.0375010	772.3021	1211.5978
BCDIN3D-AS1	0.5604	0.00212628	0.0397839	119.1647	204.7264
DHRS4-AS1	0.5573	0.00215559	0.0400524	52.0089	94.6170
UST-AS1	0.3628	0.00217561	0.0402861	14.8251	36.3555
ANKRD36BP1	0.5118	0.00222347	0.0407881	61.5043	120.2720
ASH1L-AS1	0.3091	0.00230484	0.0421312	3.5287	13.0423
LINC00692	0.2275	0.00257105	0.0455473	5.5084	25.1891
LINC00273	0.2419	0.00271628	0.0472559	3.5277	26.8782

**Figure 1:**
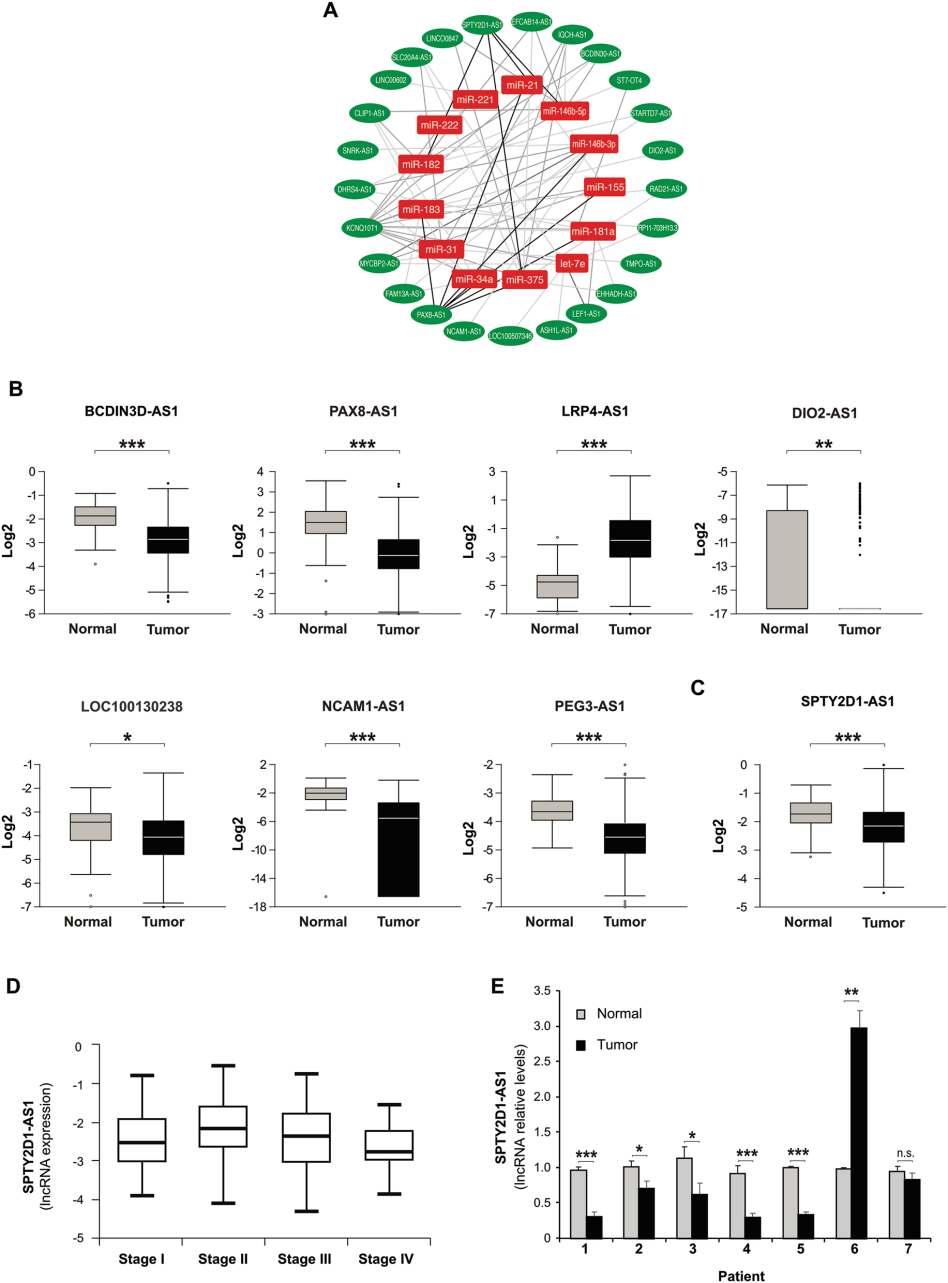
RNA sequencing unveils a network between lncRNAs and miRNAs in PTC and reveals SPTY2D1-AS1 as an lncRNA downregulated in thyroid cancer. (**A**) Core miRNA–lncRNA regulatory network involving downregulated lncRNAs predicted to target key upregulated miRNAs in papillary thyroid cancer. Using the DIANA-LncBase v2.0 to predict miRNA–lncRNA interactions, we determined which lncRNAs among the 44 downregulated in a previous study^7^ contain putative miRNA responsive elements for the top upregulated thyroid cancer oncomiRNAs. The miRNA–lncRNA network drawn by Cytoscape 3 shows the relationships between 25 lncRNAs (green ovals) and the miRNAs they are predicted to regulate (red squares). (**B**,**C**) Expression levels of the indicated lncRNAs in normal and tumor tissues obtained from non-paired 59 normal and 497 tumor samples from The Cancer Genome Atlas (TCGA) database (www.tanric.org). (**D**) SPTY2D1-AS1 levels in tumor tissues subdivided by stage (I–IV). Data were obtained from TCGA database (www.tanric.org). (**E**) Sequencing assay validation: RT-qPCR of SPTY2D1-AS1 levels in three technical replicates were performed on an independent cohort of matched human tumor and non-tumor samples (n = 7). Values represent the relative change in expression levels. Values represent mean ± SD. (*p < 0.05, **p < 0.01, ***p < 0.001).

To identify the most relevant miRNA-lncRNA interactions, we limited our search to those miRNAs that were significantly deregulated in the same samples. In a previous study, we had already identified 13 upregulated and 8 downregulated after pairwise comparison in those samples^7^. Thus, we next searched for interactions between deregulated lncRNAs and miRNAs using DIANA-lncBASE v2.0, an experimentally supported database that predicts MREs on lncRNAs^28^. Figure 1A shows the predicted interactome between 25 downregulated lncRNAs and 13 upregulated miRNAs in our samples. Strikingly, one of the downregulated lncRNAs, SPTY, contained several putative MREs with high scores for four of the most upregulated and abundant miRNAs in PTC (miR-146-5p, miR-182, miR-21, and miR-375). We therefore focused our study on SPTY as a potential tumor suppressor lncRNA.

To validate our RNA-seq results, we interrogated the TCGA database and performed a differential analysis of 59 tumor-normal pairs followed by a correlation analysis on 497 PTC tumors^6^. We selected the six most deregulated lncRNAs and, in accordance with our results, we observed the downregulation of DIO2-AS1 (fold change (FC) = 0.13), LOC100130238 (FC = 0.17), NCAM1-AS1 (FC = 0.26), PEG3-AS1 (FC = 0.23) and PAX8-AS1 (FC = 0.38), BCDIN3D-AS1 ([FC] = 0.57), and the upregulation of LRP4-AS1 (FC = 14.5) (Fig. 1B). Importantly, we also observed a downregulation of our selected candidate, SPTY, in TCGA cohort (FC = 0.79) (Fig. 1C). Of note, SPTY was more downregulated in the most advanced tumors, categorized as stage IV (Fig. 1D). In addition, we also studied the expression of SPTY in different tumor types by interrogating the TCGA database. SPTY is also downregulated in cervical squamous cell carcinoma and endocervical adenocarcinoma, head and neck squamous cell carcinoma, kidney chromophobe, kidney renal clear cell carcinoma and kidney renal papillary cell carcinoma (Supplementary Fig. S1). Finally, we performed qRT-PCR analysis of SPTY in an independent set of PTC samples, which confirmed that SPTY was downregulated in 6 of 7 PTC tumors (Fig. 1E).

### SPTY is predicted to impair the biogenesis of oncogenic miRNAs

**Figure 2:**
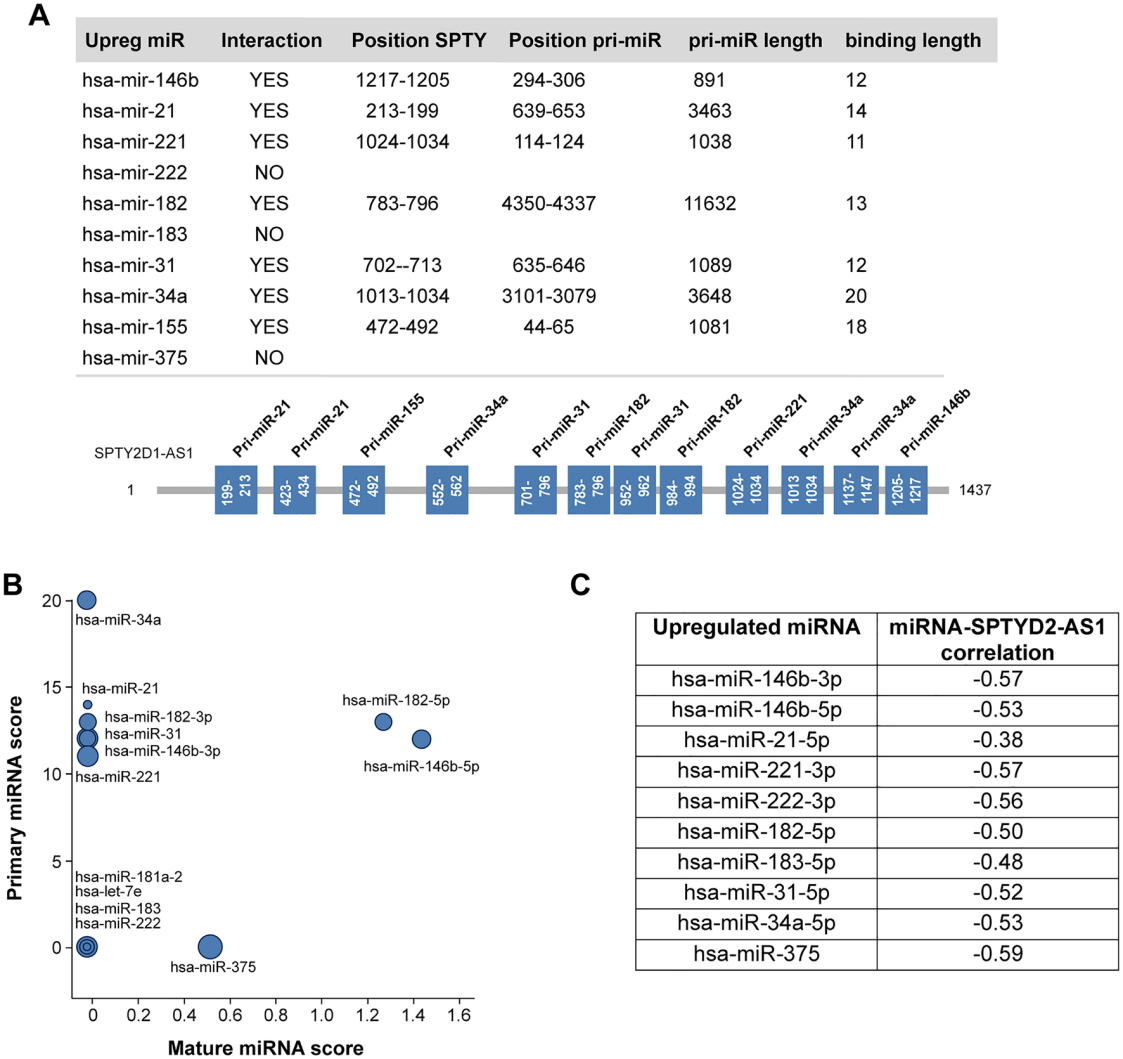
SPTY2D1-AS1 is predicted to impair the biogenesis of oncogenic miRNAs. (**A**) SPTY2D1-AS1 is predicted to target key oncogenic pri-miRNAs. Upper panel: Using Blast for sequence alignment (as described in Ref 29), we determined that SPTY2D1-AS1 putatively binds to the indicated pri-miRNAs. Lower panel: Schematic representation of pri-miRNA binding sites in the SPTY2D1-AS1 sequence. (**B**) Potential matches (based on sequence complementarity) between pri-miRNAs and mature miRNAs sequences and the lncRNA SPTY2D1-AS1. (**C**) Correlation of SPTY2D1-AS1 and the indicated miRNAs expression in PTC tumors.

There is evidence that lncRNAs may also regulate pri-miRNA processing^29^. We searched for potential complementary regions between SPTY and the stem-loop sequence of the main 10 pri-miRNAs upregulated in thyroid cancer using NCBI Blast (http://www.ncbi.nlm.nih.gov/blast). The analysis revealed that SPTY putatively binds to 7 pri-miRs: pri-miR-146b, pri-miR-21, pri-miR-221, pri-miR-182, pri-miR-31, pri-miR-34a and pri-miR-155 (Fig. 2A,B). Analysis of the cohort of 8 matched PTC tumors and normal tissue showed a negative correlation of expression between SPTY and the 10 upregulated miRNAs (Fig. 2C). Overall, using two different homology-based algorithms, SPTY was predicted to interact with the mature and primary forms of multiple upregulated miRNAs in thyroid cancer, including the three most upregulated and oncogenic miRNAs—miR-146b, miR-21, and miR-221—making this lncRNA a strong candidate for a tumor suppressor.

### SPTY decreases cell viability, migration, and invasion in vitro

**Figure 3:**
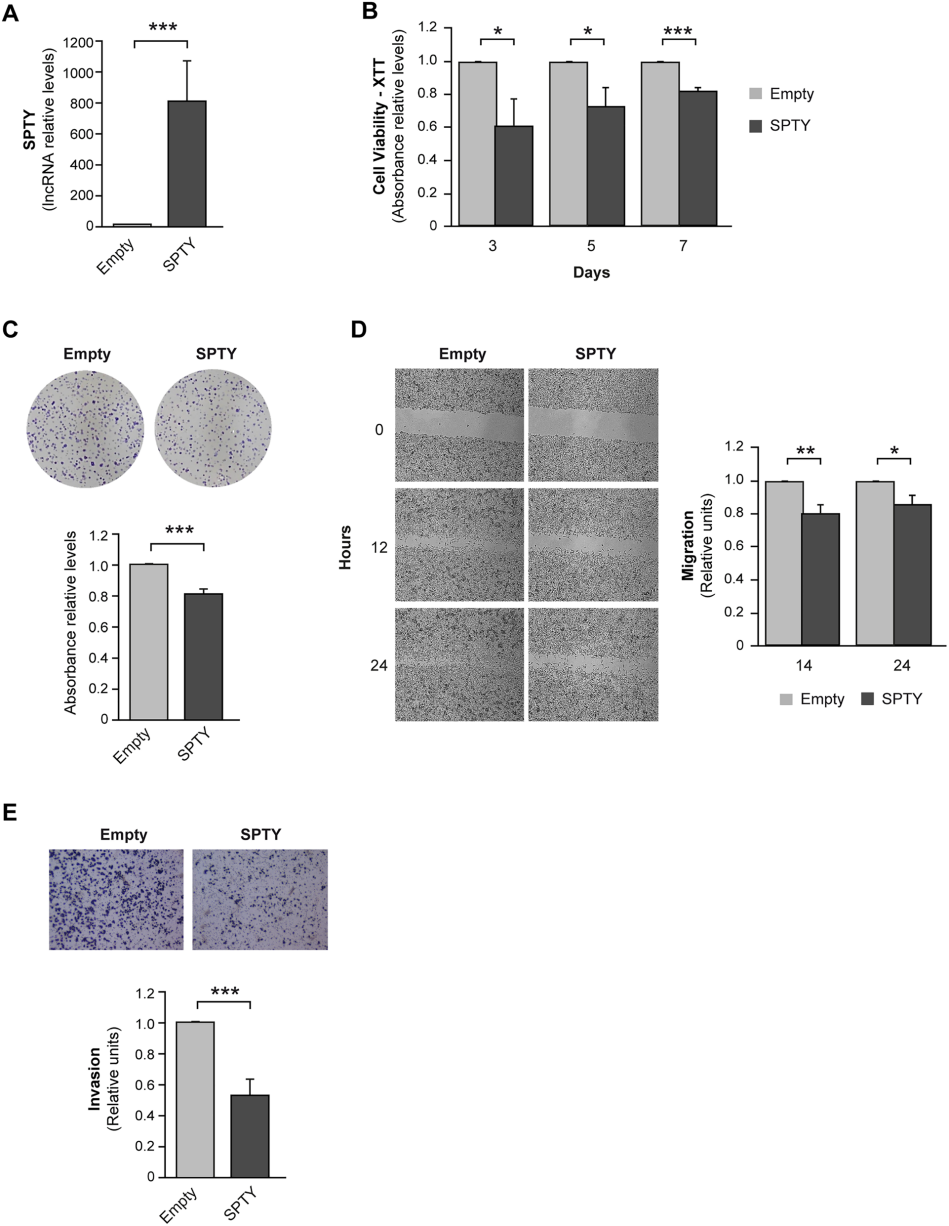
SPTY2D1-AS1 overexpression suppresses cell viability, migration, and invasion in vitro. Cal62 cells were transfected with an SPTY2D1-AS1 (SPTY) expression vector or the empty vector (**A**) SPTY levels relative to the empty vector-transfected cells after 72 h of transfection. (**B**) XTT cell viability assay at the indicated times points. (**C**) Upper panel: representative images of crystal violet-stained colonies. Bottom panel: quantification of crystal violet absorbance. (**D**) Migration assay. Left panel: representative images of a wound-healing assay. Right panel: quantification at the indicated time points after scratching. (**E**) Cell invasion assay. Upper panel: representative images of the lower chamber (invading cells). Bottom panel: cell invasion quantification relative to empty vector-transfected cells. Values represent mean ± SD. *p < 0.05; **p < 0.01; ***p < 0.001.

To investigate the role of SPTY in thyroid cancer, we first analyzed the expression levels of the lncRNA in a panel of 12 human thyroid cancer cell lines. All of them showed a strong downregulation of SPTY when compared to a normal thyroid cell line (Supplementary Fig. S2). We selected the cell line Cal62 as it represents a good model to study thyroid cancer cell behavior in vitro and in vivo as shown in previous studies of our group^12^. We next performed several gain-of-function in vitro assays in Cal62 overexpressing SPTY. Transient transfection of the SPTY vector considerably and significantly increased SPTY levels in Cal62 cells compared with control (empty vector-transfected) cells (Fig. 3A). SPTY overexpression significantly compromised Cal62 cell viability and proliferation measured by an XTT reduction assay (Fig. 3B) and by crystal violet staining (Fig. 3C). We also studied the effects of SPTY over-expression on migration and invasion capacity—two of the main hallmarks of cancer cells. Cal62 cells overexpressing SPTY migrated (Fig. 3D) and invaded (Fig. 3E) less efficiently than control cells in wound healing and Matrigel Transwell assays, respectively. Overall, these findings suggest that transient overexpression of SPTY in Cal62 cells leads to a less aggressive phenotype, supporting its function as a tumor suppressor.

### SPTY decreases tumor growth in vivo

**Figure 4:**
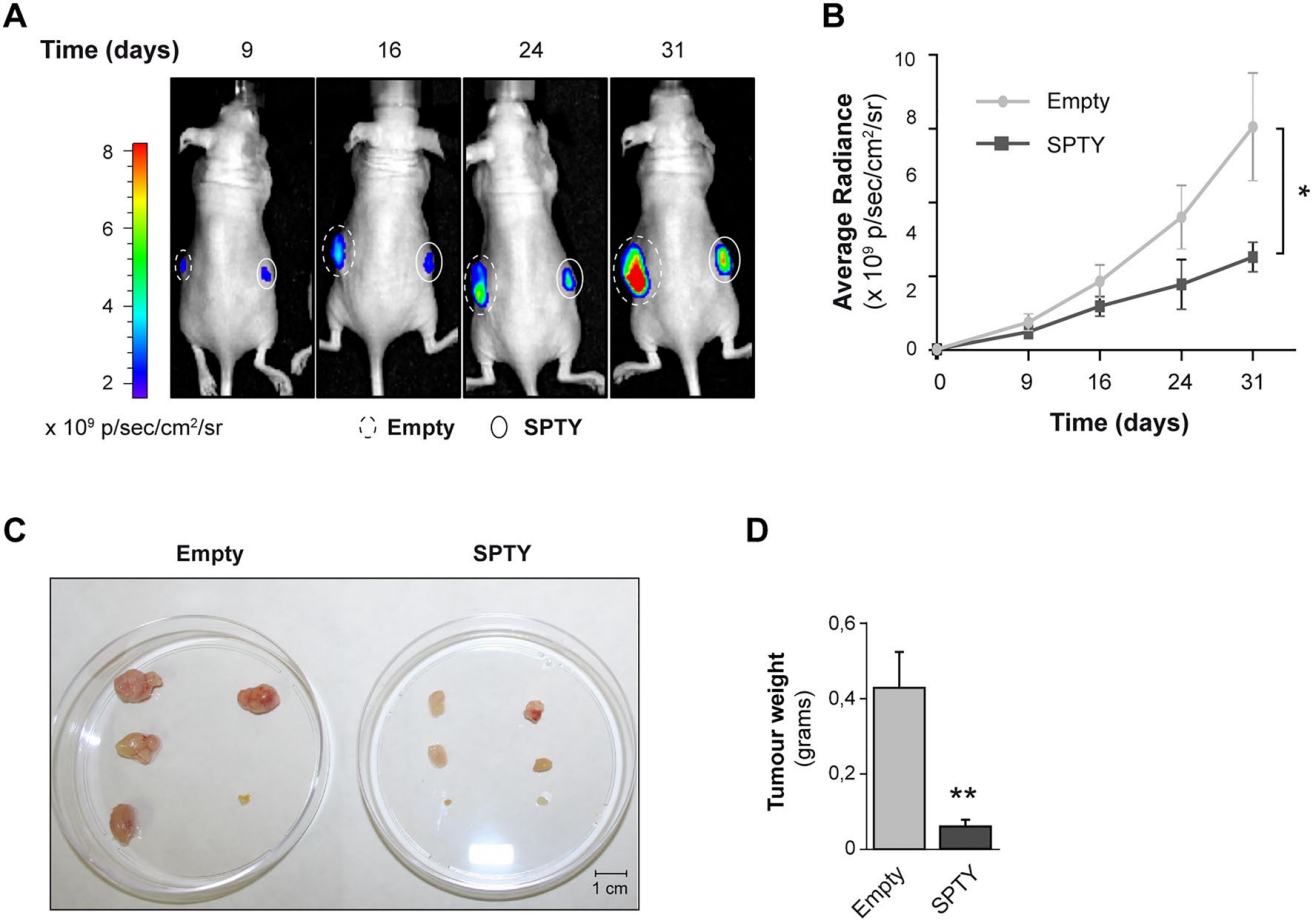
SPTY2D1-AS1 overexpression decreases tumor growth in vivo. Xenograft tumors were generated by subcutaneous injection with Cal62-Luc cells overexpressing SPTY2D1-AS1 (SPTY) or control (empty) vector. (**A**) Representative images of the bioluminescent signal of the generated tumors at the indicated time points. (**B**) Tumor radiance quantification at the indicated time points. (**C**) Generated tumors after excision. (**D**) Tumor weight of the generated tumors. Values represent mean ± SEM. *p < 0.05; **p < 0.01.

To question whether the upregulation of SPTY expression in aggressive thyroid cancer cells impacts tumor growth in vivo, we performed xenograft studies in nude mice using Cal62-luc cells stably expressing SPTY or its empty vector. Tumors of Cal62-luc cells were monitored for growth at different time points using the IVIS-Lumina II imaging system. As anticipated by the in vitro results, tumor growth was significantly suppressed in the SPTY overexpressing group relative to the control group (Fig. 4A–C). At the final time point (day 31), we dissected the xenograft tumors and measured tumor weight. As shown in Fig. 4D, tumor weight was significantly lower in the SPTY overexpressing group than in the control group. Taken together, the in vitro and in vivo gain-of-function experiments establish that SPTY acts as a tumor suppressor lncRNA in thyroid cancer.

### SPTY reduces the expression of the thyroid oncomiRNA hsa-miR-221

**Figure 5:**
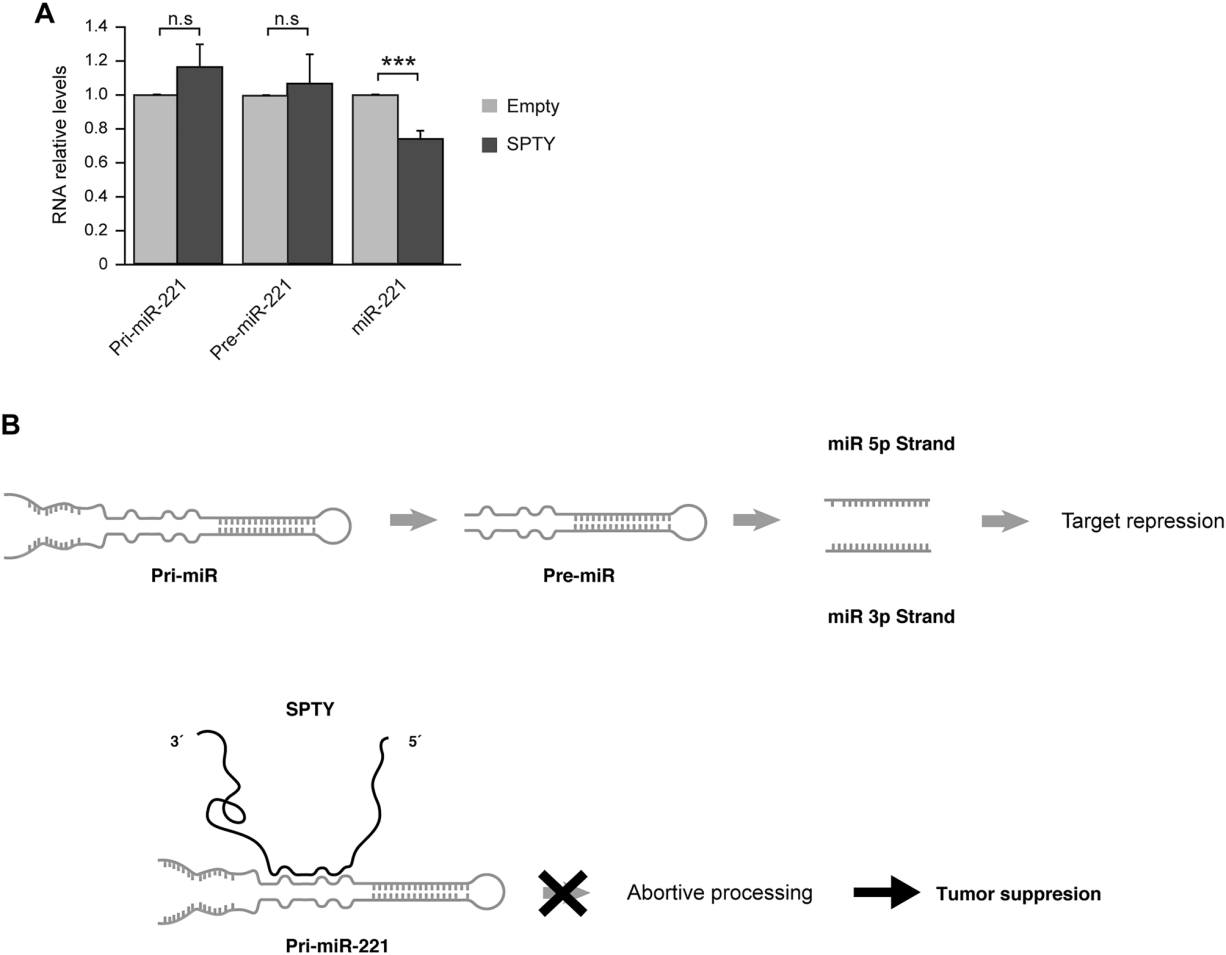
SPTY2D1-AS1 as a miRNA sponge of pri-miR-221. (**A**) pri-miR, pre-miR and miR-221 levels in Cal62 cells overexpressing SPTY2D1-AS1 (SPTY) relative to the empty vector-transfected control cells. (**B**) Schematic summary. SPTY2D1-AS1 is predicted to bind to pri-miR-221, disrupting miRNA biogenesis processing and decreasing the levels of mature miR-221. As miR-221 is considered an oncomiRNA, its downregulation may result in the tumor suppressive features of SPTY2D1-AS1 expression. Values represent mean ± SD. ***p < 0.001, *n.s.* not significant.

SPTY is predicted to interact with the mature and primary forms of multiple upregulated miRNAs in thyroid cancer, including the three most upregulated and oncogenic miRNAs: miR-146b, miR-21, and miR-221 (Figs. 1A, 2A,B). We thus used SPTY-overexpressing Cal62 cells to study the levels of the pri-miRNA, pre-miRNA, and mature forms of these oncomiRNAs. SPTY overexpression did not affect the levels of the mature forms of miR-146b and miR-21 (results not shown), but it significantly reduced the levels of mature miR-221 (Fig. 5A). The levels of the pri- and pre-miR-221 forms did not change in SPTY-overexpressing cells, suggesting that SPTY binds to the primary form of miR-221, blocking its processing to the mature form (Fig. 5A). Taken together, our findings suggest that SPTY acts as a tumor suppressor lncRNA by reducing the levels of the miR-221 oncomiRNA.

## Discussion

In this work, we investigated differentially expressed lncRNAs in PTC and explored their interactions with differentially expressed miRNAs in paired thyroid tumor and non-tumor samples. Using two different homology-based algorithms, we identified a likely operative post-transcriptional regulatory network in which the downregulated lncRNA, SPTY, is anticipated to target many of the most abundant and upregulated miRNAs in thyroid cancer. Although predicted to operate through the sponging of the mature forms of miRNAs, we found that it likely blocks the processing of the primary form of miR-221, an important upregulated oncomiRNA in many tumor types^13–17^, including thyroid cancer^6^. We also show that SPTY functions as a potent tumor suppressor in vitro and in vivo and is downregulated in the most advanced stages of human PTC.

LncRNAs have emerged as key regulators of cancer pathways and as biomarkers of disease^30^. Although tens of thousands of lncRNAs have been identified by high- throughput RNA sequencing, only a small percentage of these have been functionally characterized through differential expression analysis and comparative transcriptomic studies of cancer specimens. Various lncRNAs have been shown to have important biological functions and serve important roles in gene regulation, revealing a diversity of phenotypes and mechanisms^31^. Some abundant lncRNAs affect gene expression by functioning as competitive endogenous RNAs (ce-RNAs)^32,33^. This hypothesis proposes that some RNA molecules with shared microRNA (miRNA) binding sites compete for post- transcriptional control, leading to diminished target gene repression. Such RNA molecules reduce miRNA availability to target mRNAs and lncRNA have been shown to play a major role. Indeed, the use of an experimental lncRNA that sponge miRNAs has been shown to artificially derepress miRNA targets as effectively as antisense oligonucleotides^34–36^. Our study is the first to fully explore the lncRNA–miRNA interactions in thyroid cancer. Through RNA sequencing (RNA-seq) of paired normal and tumor thyroid samples from 8 patients with PTC, we uncover the main differentially expressed lncRNAs and their interactions with common differentially expressed miRNAs in PTC. Because many lncRNA have tissue-specific and cancer-specific expression patterns, our results provide potential biomarkers and a rationale to target them clinically in a thyroid cancer context.

In the present study, SPTY, a lncRNA specifically suppressed in thyroid cancer, was predicted to compete for important thyroid cancer-related miRNAs such as miR-221. Interestingly, based on our bioinformatic analysis, such an effect seems to be mediated through the impairment of miRNA biogenesis. MiRNA biogenesis is a multilayered process in which transcription of miRNAs produces a long primary molecule (the pri-miRNA) that folds into hairpins and is a substrate for DROSHA, which releases a 70-nt pre-miRNA that is exported to the cytoplasm and further cropped by DICER1 into the mature form^37^. Thus, pri-miRNAs are nuclear species that undergo DROSHA cleavage before being exported to the cytoplasm. Likewise, there is evidence that lncRNAs can be retained in the nucleus and play important functions there^31^. In the seminal paper by Liz et al., a lncRNA that is mainly expressed at the nucleus can directly interact and regulate pri-miRNA maturation at the level of DROSHA processing^29^. Such regulation requires complementarity between the lower stem region of the pri-miRNA transcript and a sequence in the lncRNA, impairing microprocessor recognition and efficient pri-miRNA cropping.

In general, ceRNAs act as miRNA sponges and reduce the activity of the target miRNAs essentially without altering their biogenesis^34^. However, our results suggest a negative regulation of pri-miRNA processing that depends on direct RNA-RNA interaction between the stem-loop sequence of the pri-miRNA and SPTY (Fig. 5B). According to the in silico analysis, the number of potential complementary regions predicts a strong interaction between the lower stem region of pri-miR-221 and a sequence present in the region of the lncRNA SPTY (Fig. 5B). Although we cannot exclude alternative regulatory mechanisms, our finding supports a previous model described by Liz et al. in which disruption of the lower stem structure through lncRNA strand invasion prevents optimal recognition of the region and consequently blocks efficient processing by DROSHA^29^. In addition, our in silico analysis revealed that SPTY might regulate other important thyroid cancer-related miRNAs, although this awaits further investigation.

Several lncRNAs have been linked to thyroid cancer, yet the mechanisms through which they act remain unclear. The first lncRNAs implicated in thyroid cancer, termed papillary thyroid carcinoma susceptibility candidate 1–3 (PTCSC1, PTCSC2, and PTCSC3), are involved in PTC predisposition and are significantly down-regulated in thyroid tumors, implying roles as tumor suppressors^38–40^. Another example is NAMA, a lncRNA that is linked to the MAP kinase pathway and growth arrest and it is highly associated with the activating BRAF mutation V600E in PTC^41^. The association of NAMA and other lncRNAs with BRAF was further confirmed by RNA-seq-based analysis^42^. Additionally, some lncRNAs have been associated with a worse clinical outcome and are considered as prognostic markers^43,44^. However, there are very few examples of miRNA–lncRNA competitive interactions experimentally proven in thyroid cancer^45,46^. Of these, one of particular interest is Klhl14-AS. This lncRNA targets two upregulated miRNAs in thyroid cancer, miR-182-5p and miR-20a-5p, which silence essential players of thyroid differentiation and apoptosis including PAX8 and BCL2^47^.

Our work has some limitations related to the nature of lncRNAs. First, skepticism remains about whether physiological expression levels of a single lncRNA, which can represent a small fraction of the total miRNA targets, is sufficient to alter miRNA regulation^30^. For this reason, the abundance of a particular lncRNA species appears to be a key indicator of the mechanism by which it exerts its function, thereby requiring careful evaluation^31^. In this sense, SPTY has an intermediate-high abundance in our human samples according to the RNA-sequencing data when compared to other differentially expressed lncRNA (around 192.8508 mean RPM)(see Table 2, grey-bold mark). The second limitation is related to the homology-based prediction methods for establishing lncRNA-miRNA interactions, which can vary significantly between algorithms^48^. Moreover, sequence content alone does not take into account the physiological expression levels of the ceRNA, miRNA and target genes. A strategy to partially overcome this limitation is to prioritize ceRNAs displaying biochemical enrichment with RNA-induced silencing complex (RISC) components, which is the case in the algorithm that we used, DIANA-lncBASE v2.0^28^. Third, although gene expression data across patient cohorts can help to prioritize lncRNAs that have a negative expression correlation with target miRNAs (see Fig. 2C), it does not discriminate between correlated genes owing to alternative regulatory mechanisms. Nonetheless, the potential complementary regions between SPTY and the stem-loop sequence of 7 pri-miRNAs upregulated in thyroid cancer provide an opportunity for hypothesis generation, suggesting that miRNA biogenesis could be impaired^29^. This seems to be the case for miR-221, but not for miR-146b and miR-21, according to our experimental data. SPTY overexpression in thyroid cancer cells did not affect the levels of the mature forms of miR-146b and miR-21 (results not shown), but it significantly reduced the levels of mature miR-221. We are currently performing mutagenesis of the primary form of this miRNA and studying target genes expression to fully demonstrate our findings.

Finally, miR-221 has various functions in biological systems^49^ and has been described as an oncomiRNA in several tumor types^13–17^. MiR-221 is one of the most upregulated miRNAs in thyroid cancer^6,7^, and it has been demonstrated to target p27kip1^19^, a key regulator of cell cycle. Moreover, miR-221 has a potential role as a prognostic biomarker for recurrence in PTC^21^ and as a serum marker for the follow-up of thyroid cancer^50^. Thus, miR-221 could play a crucial role in future innovative therapeutic strategies^51^. Models of competitive binding between lncRNAs and miRNAs have been frequently related to invasion, migration and EMT in multiple cancer types that are supported by in vivo experiments in the context of metastases^30^. Similarly, we believe that SPTY, an experimentally proven tumor suppressor in vitro and in vivo (Figs. 3 and 4), competitively binds to the miR-221 resulting in upregulation of target genes involved in tumor suppressive activities that we are currently investigating.

In summary, we report on an interaction network between lncRNAs and miRNAs that might be operative in PTC and provide evidence that it has functional consequences. We show that the lncRNA SPTY has tumor suppressor activity and is selectively downregulated in thyroid cancer. In addition, we describe that SPTY could alter miRNA biogenesis of important oncogenic miRNAs such as miR-221, pointing to the potential application of SPTY in the treatment of thyroid cancer.

## Methods

### Patients

Samples of PTC tumors and contralateral normal thyroid tissue from the same patients (n = 8) used for the RNA-seq analysis were obtained from the La Paz University Hospital Biobank (Madrid, Spain). The main clinical characteristics of the patients have been described^7^. An independent set of PTC tumors (n = 7) was used for quantitative reverse-transcription PCR (qRT-PCR) validation studies and was also obtained from the same Biobank. The main clinical characteristics of the second cohort of patients have been defined previously^52^. Informed consent was obtained from all the patients following the protocols approved by the Bioethics Committee at the Hospital Universitario La Paz (*Comité Ético de Investigación Clínica (CEIC) del Hospital Universitario La Paz).* This study was conducted according to the guidelines of the Declaration of Helsinki.

### Next-generation sequencing

The sequencing procedure was carried out on the Genome Analyzer IIx Platform (Illumina) at the Genomics Core Unit of the Spanish National Cancer Research Center, Madrid) using protocols recommended for mRNA-seq (Illumina TruSeq Stranded mRNA). The mRNA reads were aligned to the human genome (UCSC, hg19 assembly) using TopHat v.2.0.4, permitting two mismatches and a maximum of five multi-hits. Gene-level expression was calculated as the sum of all read counts over their exons using Htseq-count and the gene annotation from the reference genome (hg19). All sequencing data can be downloaded from the Gene Expression Omnibus (GEO) under accession number GSE63511, and more details are given in our previous study^7^.

### MiRNA:lncRNA in silico predicted interactions

In silico target prediction for human and mouse spliced lncRNA sequences was performed with the DIANA-microT algorithm, appropriately adjusted. miRNA Recognition Elements (MREs) were scored separately and each miRNA:lncRNA interacting pair was characterized by a cumulative score that signifies the interaction strength according to DIANA-LncBASE v2.0^28^. Potential stem-loop sequence RNA-RNA interaction sites in pri-miRNAs were obtained through the alignment of two nucleotide sequences in NCBI BLAST (http://www.ncbi.nlm.nih.gov/blast)^53^. Through this tool we obtained local alignments scores between RNAs, considering penalties for gaps and substitutions, using non-conservative approach—i.e. no megablast optimization—Predicted contacts in the stem-loop regions of the pri-miRNAs were checked for correct strand homology and length.

### Bioinformatic analysis of the cancer genome atlas

The expression levels of the lncRNAs DIO2-AS1, LOC100130238, NCAM1-AS1, PEG3-AS1, BCDIN3D-AS1, PAX8-AS1, LRP4-AS1 and SPTYD1-AS1 in normal and PTC tissues were obtained from the RNA-seq dataset of TCGA using the TANRIC (https://www.tanric.org) web resource.

### Cell culture and transfections

The human ATC cell line Cal62 was grown in Dulbecco's modified Eagle's medium (DMEM) supplemented with 10% fetal bovine serum (FBS). Transfection assays were performed using Lipofectamine 2000 with OptiMEM medium (both from Thermo Fisher). The SPTY sequence was cloned into the pcDNA3.1 expression vector (Thermo Fisher), which was also utilized as the control (empty vector) in all transiently transfection assays. For xenograft experiments we used Cal62 expressing a vector harboring luciferase and GFP, CMV-Firefly Luc-IRES-EGFP, which was constructed by Dr J. Blanco (IQAC-Consejo Superior de Investigaciones Científicas [CSIC], Barcelona, Spain). Cal62 cells stably expressing this construct (termed Cal62-Luc) were generated and kindly provided by Dr Eugenia Mato (IIB, Sant Pau, Barcelona, Spain) and then we stably transfected these cells with pcDNA3.1 expressing SPTY (selected with neomycin).

### RNA quantification

Total RNA was isolated from cells with Trizol Reagent (Invitrogen). RT-PCR assays for SPTY and miRNAs were performed using the M-MLV Reverse Transcriptase Kit (Promega Corporation) or with NCode™ miRNA First-Strand cDNA Synthesis and the qRT-PCR Kit (Invitrogen), respectively. qRT-PCR was performed with the Kapa Sybr Fast Universal Kit from Sigma-Aldrich. All primers were purchased from Sigma-Aldrich and are described in Supplementary Table I.

### Viability assays

Cell proliferation assays using the tetrazolium XTT compound were performed using a kit from Canvax. Transfected Cal62 thyroid cells were seeded in 96-well plates and measurements were performed in a spectrophotometer after 3, 5 and 7 days. Cell viability was also assessed by crystal violet staining of 2,500 Cal62 cells seeded in each well of a 6-well plate, 24–48 h after transfection. Cells were fixed in 4% formaldehyde after 7–10 days and stained with crystal violet. After extensive washing and drying, crystal violet was resolubilized in 10% acetic acid and quantified at 590 nm as an indirect measure of cell number.

### Migration assays

Wound healing (scratch) assays were performed with 90% confluent cell monolayers. Twenty-four hours after transfection, cells were treated for 2 h with 10 µg/mL mitomycin C in 10% FBS DMEM medium to inhibit proliferation. After treatment, monolayers were scratched with a 10-µL pipette tip and the width of the wound was measured at 0-, 12-, and 24-h using ImageJ software (NIH).

### Invasion assays

Invasion was examined in transwell cell culture chambers using 8-μm pore polycarbonate membranes coated with Matrigel on the upper side (Corning Biocoat). Cal62 cells were suspended in DMEM culture medium with 0.2% FBS. Around 3.5 × 10^4^ cells were placed in the upper chamber 48 h after transfection. The lower chamber contained 0.75 mL of medium with 20% FBS as a chemoattractant. Cells were allowed to invade to the lower chamber, and the cells that remained on the upper chamber were removed with a cotton swab; filters were fixed in 4% paraformaldehyde and stained with crystal violet. Using image J, five fields were quantified for each condition and the number of cells migrated to the lower surface was counted.

### In vivo study

Animal experimentation was performed in compliance with the European Community Law (86/609/EEC) and the Spanish law (R.D. 1201/2005), with the approval of the CSIC ethics committee. The study was carried out in compliance with the ARRIVE guidelines. Xenotransplants were established in 6-week-old immunocompromised female Hsd:Athymic Nude-Foxn1nu mice (Envigo) by subcutaneous injection in both flanks of 1 × 10^6^ Cal62-luc cells constitutively overexpressing the SPTY expression vector (or the empty vector) and suspended in 50 μL of phosphate buffered saline mixed with 50 μl of Matrigel (Corning). In total, 11 tumors were established (Empty vector n = 5 and SPTY n = 6). Tumor bioluminescent signals were determined in vivo at the indicated time points to calculate tumor growth. To do this, 50 μL of a 40 mg/mL solution of Xeno-Light D-Luciferin- K + Salt Bioluminescent Substrate (Perkin Elmer) was subcutaneously injected into each mouse at each time point. At 10 min post-injection, mice were anesthetized and imaged with the IVIS-Lumina II Imaging System (Caliper Life Sciences). At the final time point (day 31), tumors were excised and weighed.

### Statistical analysis

Results are expressed as the mean ± SD of at least three different experiments performed in triplicate. Results from the in vivo studies are expressed as the mean ± SEM. Statistical significance was determined by Student's t-test analysis (two-tailed) and differences were considered significant at a P-value < 0.05.

### Ethical approval

All authors declare that not competing financial interests and non-financial interest exist. The animal in vivo experiments have been performed in accordance with the ARRIVE guidelines.

## Supplementary Information

Supplementary Figure 1.

Supplementary Figure 2.

Supplementary Table 1.

Supplementary Legends.
