# Supplementary figures and images for: Identification of an interactome network between lncRNAs and miRNAs in thyroid cancer reveals SPTY2D1-AS1 as a new tumor suppressor
Source: Sci Rep. 2022 May 11;12:7706. doi: 10.1038/s41598-022-11725-4 (PMC9095586; doi:10.1038/s41598-022-11725-4)

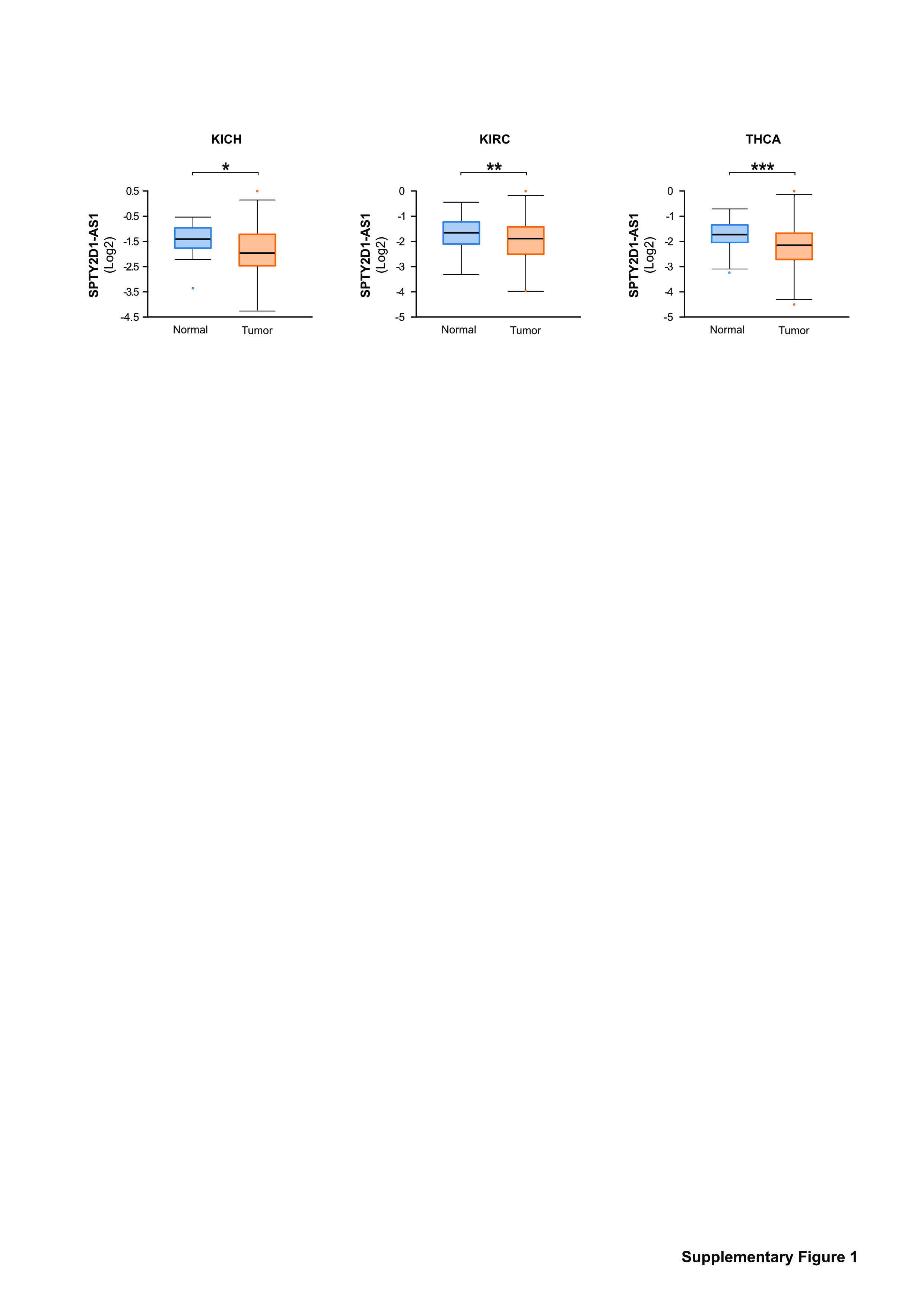

Supplement: Supplementary file 1 — Supplementary Figure 1. [file 41598_2022_11725_MOESM1_ESM.tiff]

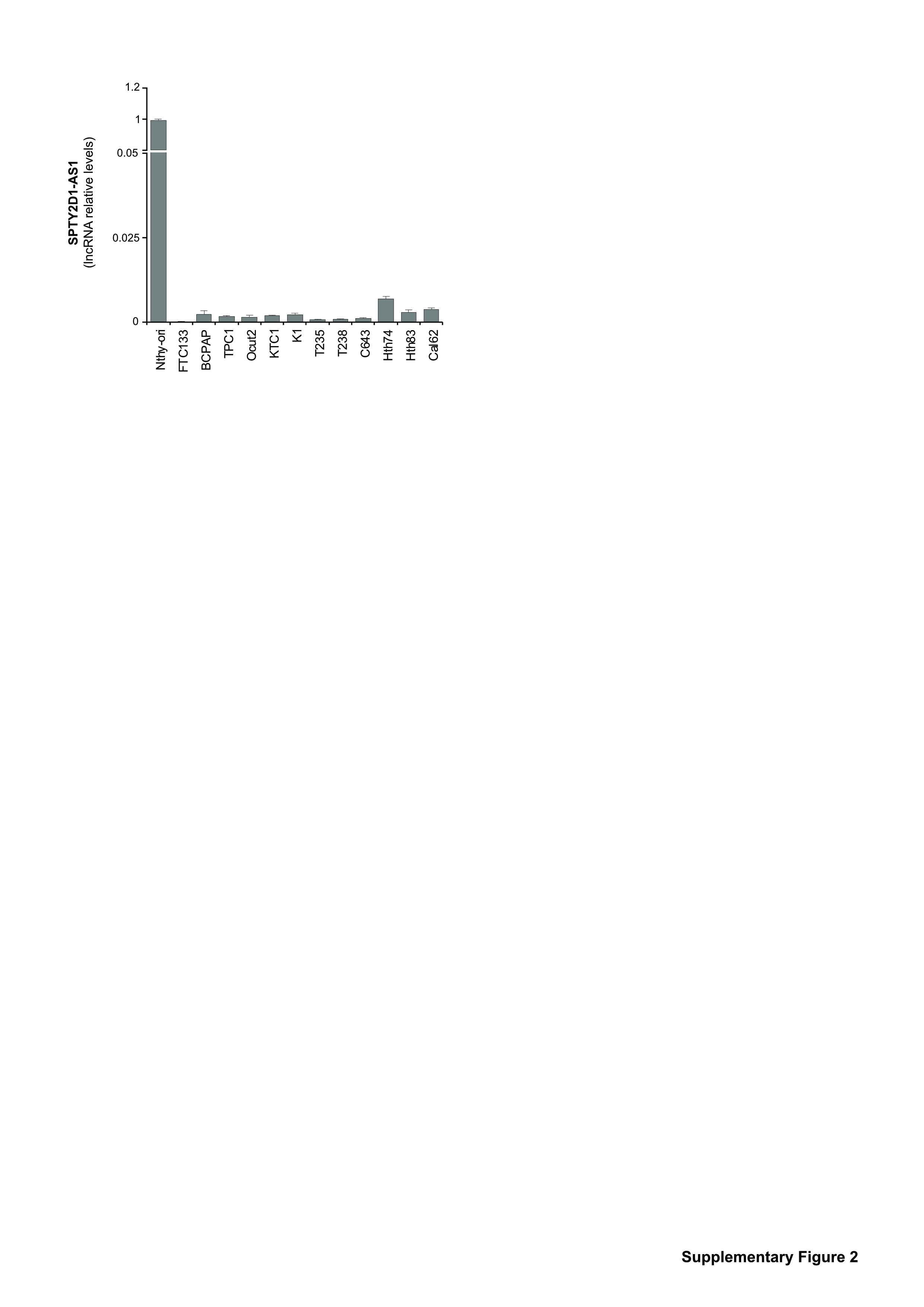

Supplement: Supplementary file 2 — Supplementary Figure 2. [file 41598_2022_11725_MOESM2_ESM.tiff]
