# Supplementary material for: Identification of an interactome network between lncRNAs and miRNAs in thyroid cancer reveals SPTY2D1-AS1 as a new tumor suppressor
Source: Sci Rep. 2022 May 11;12:7706. doi: 10.1038/s41598-022-11725-4 (PMC9095586; doi:10.1038/s41598-022-11725-4)
Supplement: Supplementary file 3 — Supplementary Table 1. [file 41598_2022_11725_MOESM3_ESM.docx]

**Supplementary Table I: Primers sequence used to amplify the main genes.**

|  | **Forward primer** | **Reverse Primer** |
| --- | --- | --- |
| **SPTY** | GCCCTTCGACGTTGCATTTC | GGCATTTTGAGAGCATCAGCA |
| **Pri-miR-221** | ACTTGCAAGCTGAACATCCA | CCTGAAACCCAGCAGACAAT |
| **Pre-miR-221** | TGAACATCCAGGTCTGGGGCAT | GAGAACATGTTTCCAGGTAGCCT |
| **miR-221** | AGCTACATTGTCTGCTGGGTTTC |  |
