# Supplementary material for: Identification of an interactome network between lncRNAs and miRNAs in thyroid cancer reveals SPTY2D1-AS1 as a new tumor suppressor
Source: Sci Rep. 2022 May 11;12:7706. doi: 10.1038/s41598-022-11725-4 (PMC9095586; doi:10.1038/s41598-022-11725-4)
Supplement: Supplementary file 4 — Supplementary Legends. [file 41598_2022_11725_MOESM4_ESM.docx]

**Identification of an interactome network between lncRNAs and miRNAs in thyroid cancer reveals SPTY2D1-AS1 as a new tumor suppressor**

Julia Ramírez-Moya^1,2,#,^ León Wert-Lamas^1,#^, Adrián Acuña-Ruíz^1,2^, Alice Fletcher^1,3^, Carlos Wert-Carvajal^1,4^, Christopher J McCabe^3^, Pilar Santisteban^1,2*^ and Garcilaso Riesco-Eizaguirre^1,2,5,6*^

1. Instituto de Investigaciones Biomédicas “Alberto Sols”, Consejo Superior Investigaciones Científicas, and Universidad Autónoma de Madrid (CSIC-UAM), E-28029 Madrid, Spain. jramirez@iib.uam.es (J.R-M); Leon_WertLamas@dfci.harvard.edu (L.W-L); aacuna@iib.uam.es (A.A-R); carloswertcarvajal@gmail.com (C.W-C).

2. Centro de Investigación Biomédica en Red de Cáncer (CIBERONC), Instituto de Salud Carlos III (ISCIII), E-28029 Madrid, Spain

3. Institute of Metabolism and Systems Research, University of Birmingham, Birmingham, B152TT United Kingdom. fletcher.alice@hotmail.co.uk (A.F); c.j.mccabe.med@bham.ac.uk (C.J.M).

4. Department of Bioengineering and Aerospace Engineering. Universidad Carlos III, E-28911 Madrid, Spain.

5. Departamento de Endocrinología y Nutrición, Hospital Universitario de Móstoles, E-28223 Madrid, Spain.

6. Universidad Francisco de Vitoria, Madrid, Spain.

# J.R-M and L.W-L contributed equally to this work and both should be considered first authors

*Correspondence: psantisteban@iib.uam.es (P.S) and griesco@iib.uam.es (G.R-E).

**Supplementary Figure legends**

**Supplementary Figure 1.** Downregulation of SPTY2D1-AS1 in tumor types other than thyroid cancer. Expression levels of SPTY2D1-AS1 in normal and tumor tissues of different tumor types obtained from The Cancer Genome Atlas (TCGA) database ([www.tanric.org](http://www.tanric.org)). Kidney chromophobe (KICH), kidney renal clear cell carcinoma (KIRC) and thyroid carcinoma (THCA). Values represent the relative change in expression levels. Values represent mean ± SD. (*p<0.05, **p<0.01, ***p<0.001)

**Supplementary Figure 2**. SPTY2D1-AS1 is downregulated in 12 human thyroid cancer cell lines. RT-qPCR of SPTY2D1-AS1 levels were performed in 12 human thyroid cancer cell lines relative to the human normal thyroid cell line Nthy-ori (named in the x axis). Values represent the relative change in expression levels. Values represent mean ± SD
